# Design and simulation of a CNTFET based approximate full adder for low power nanoelectronic and image processing applications

**DOI:** 10.1186/s11671-026-04814-0

**Published:** 2026-08-10

**Authors:** Amir Mohammad Karimi Forood, Marziyeh Narouei, Shaik Javid Basha, Shams Ul Haq, Trapti Sharma, Deepika Bansal

**Affiliations:** 1 Department of Biomedical Engineering, University of Connecticut, Storrs, CT USA; 2 Electrical and electronics engineering department, Tagliatela College of Engineering, University of New Haven, West Haven, CT USA; 3 Department of Electronics and Communication Engineering, Santhiram Engineering College, Nandyal, India; 4 Department of Electronics and Communication Engineering, Jamia Millia Islamia, New Delhi, 110025 India; 5 School of Computing Science and Engineering, VIT Bhopal University, Bhopal, Madhya Pradesh India; 6 Department of Electronics and Communication Engineering, Manipal University Jaipur, Jaipur, 303007 India

**Keywords:** CNT, CNTFET, AFA, PDP, Image blending, PVT variations

## Abstract

The unique electrical properties of carbon nanotube (CNT) materials have enabled the development of highly efficient nanoelectronic devices. Leveraging these advantages, this work presents a CNT-material-driven design of a CNTFET-based approximate full adder (AFA) optimized for low-power arithmetic operations. The proposed architecture integrates CMOS and pass-transistor logic with multi-threshold CNTFET devices to minimize voltage degradation and enhance energy efficiency. Circuit-level simulations were performed using Synopsys HSPICE with the Stanford 32-nm CNTFET model. Results demonstrate that the proposed AFA achieves a 7.2–66.5% reduction in power consumption and a 2.7–81.1% improvement in energy efficiency compared with existing designs at a 0.5 V supply. To confirm practical applicability, the AFA is incorporated into an image blending system, where it delivered superior visual fidelity based on PSNR and SSIM metrics. Comprehensive figure-of-merit analysis further confirms the correctness of the design for modern low-power nanoelectronic and approximate computing applications.

## Introduction

In digital schematics, there is a huge demand for increased processing speed and reduced power dissipation due to the rapid improvement of contemporary computation. However, a fundamental challenge arises due to the inverse relationship between dynamic power consumption and propagation delay, which makes it challenging to optimize both simultaneously [1, 2]. Innovative circuit design strategies are being investigated by researchers in order to circumvent this constraint. Approximate computation has emerged as a plausible solution among them. This technology lowers the number of FETs to make power improvements and processing time better. This betterment was acquired at the expense of introducing marginal computational inaccuracies. In the field of error-resilient applications such as multimedia processing, digital photography and machine learning algorithms, these kinds of improvements are considered and acceptable. Approximate circuits facilitate the advancement of next-generation high-speed and low-power computing architectures by maintaining a rigorous balance between efficiency and accuracy [1–6].

In the design of digital systems, adders are one of the most significant arithmetic circuits. They are the fundamental components that are utilized for performing additional functions like subtraction, multiplication, and division. The Full Adder (FA) is a vital component of a digital processor’s design, where its efficiency influences the overall performance of the system [7–9]. The rise of high-speed multimedia applications on portable devices has led to a huge need for digital circuits that can work faster and use less power. This demand necessitates substantial progress in the architectural design of the FA [10, 11]. There are several design styles, such as CMOS [12], pass transistor logic [10], transmission gate [13], dynamic circuit [14], gate diffusion input [15] and adiabatic logic [16–18], to implement the FA circuits. Furthermore, the inherent error resilience of multimedia applications presents an opportunity to develop approximate full adders (AFAs). These circuits are specifically designed to leverage this tolerance, achieving significant gains in transistor count reduction, operational speed, and power efficiency [19, 20].

From the mentioned design styles, the standard CMOS and transmission gate-based adders require a large number of transistors for the FA implementation. Whereas the pass transistor logic and gate diffusion input techniques endure with the threshold voltage drop, which makes the output levels worse [21]. In [21], it is discussed that introducing the CNTFET technology for the development of FA can improve the output full swing. Recently, the CNTFET technology attracted the researchers because of its physical structure and intrinsic electrical characteristics that resemble the CMOS technology [22, 23]. CNTFETs have a lot of promises since they have low *OFF*-current and ballistic transport behavior, which makes them work well with minimal power and high performance. Because they are comparable to MOSFETs in terms of structure and electricity, CNTFETs may be used to easily build CMOS circuit designs. This feature makes them a good choice for future VLSI technologies [24].

This paper introduces an innovative design for an AFA cell which is optimized for minimal power consumption and enhanced energy efficiency. The proposed architecture combines traditional CMOS and pass transistor logic methods in a way that reduces delay, power dissipation and overall energy usage. This architecture processes three standard inputs *A*, *B* and a carry-in (*C*) to obtain both *Sum* and *Carry* as outputs. This architecture can enhance operational speed through the concurrent computation of its outputs. Moreover, a noteworthy improvement in power consumption is achieved by minimizing the switching activity at both internal intermediate and primary output nodes.

To exhibit the efficiency of the proposed AFA, the performances, including delay, power consumption, power-delay product (PDP), transistor count, switching activity, maximum error distance (MAE), total error distance (TED), mean error distance (MED), and normalized error distance (NED), are thoroughly compared to existing AFA cells under identical simulation conditions. Then, the proposed AFA is evaluated at the application level through its deployment in an image processing task. Particularly, both the proposed and existing AFA designs are implemented within image-blending algorithms to evaluate the quality of resultant blended images. For this comparative study, major quantitative metrics such as peak signal-to-noise ratio (PSNR), the structural similarity index measure (SSIM), and figures of merit (FoM) are employed. The results show that the proposed AFA design attains superior output quality while also demonstrating improved efficiency compared to the existing designs presented in the literature.

The major contributions of this work are fivefold namely:

- Proposed a novel 11-transistor CNTFET-based AFA using a hybrid CMOS and PTL design methodology for low-power nanoelectronic applications.
- Employed multi-threshold CNTFET device engineering to minimize threshold voltage degradation and improve energy efficiency.
- Performed comprehensive robustness analysis under process, voltage, temperature (PVT), oxide thickness, CNT chirality, CNT count, and pitch variations.
- Conducted Monte Carlo simulations to verify the reliability and stability of the proposed AFA against process variations.
- Validated the practical applicability of the proposed design through an image blending application using PSNR, SSIM, and figure-of-merit (FoM) evaluations.

The remainder of this article is set up as follows. In Sect.  2, a discussion on the CNTFET device and its associated power and error is provided. A broad review of conventional AFA designs is presented in Sect.  3. The architectural design and operational principles of the proposed AFA are described in Sect.  4. Section  5 presents simulation results, key findings and a comparative study with existing works. The application of the proposed AFA in an image blending case study is discussed in Sect.  6. Section  7 concludes the study by discussing the major findings and future research directions.

## Background Information

### CNTFET Device

**Fig. 1:**
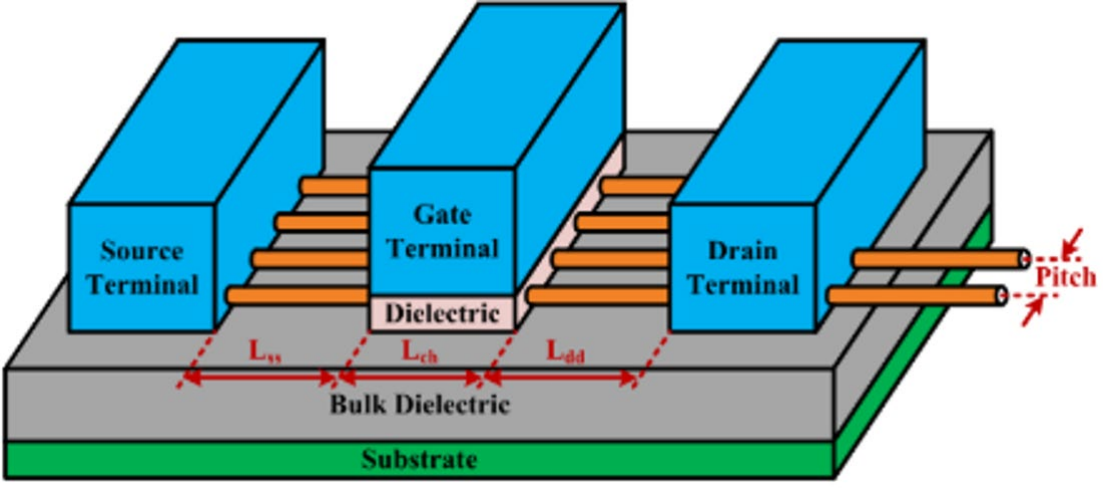
Structure of CNTFET

The CNTFET is a nanoscale transistor that utilizes one or multiple CNTs as the channel material over silicon. Due to its remarkable electrical, mechanical, and thermal characteristics, the CNTFET has become one of the most promising substitutes for traditional CMOS transistors in the nanoscale range [25]. The CNT performs better than the silicon because the quasi-one-dimensional band structure, ballistic transport capabilities, and high current carrying capacity avoid the short-channel effects and mobility loss at lower dimensions, respectively [26, 27]. Numerous studies in the literature detail the structure of CNTFETs, which are characterized by CNTs arrayed upon a thick oxide insulator layer. This structural configuration extends beyond the CNTs themselves, encompassing the device’s source, drain, and gate terminals, all of which are fabricated atop this same insulating substrate. The gate terminal includes gate oxide material with a high dielectric constant of 16 and a physical thickness of 2 to 4 nm. Conventionally, silicon dioxide (*SiO*₂) serves as the thick underlying oxide insulator, while hafnium dioxide (*HfO*₂) is employed for the high-K gate oxide [28–30]. Several additional parameters critically influence CNTFET performance, including the number of CNTs, their pitch (inter-CNT spacing), and the chirality vector (*n*, *m*). Notably, the threshold voltage (V_th_) of the device is directly determined by the diameter of the CNTs, which is itself a function of these chiral indices. The expressions to obtain the CNTFET’s *V*_*th*_ and CNT diameter (*D*_*CNT*_) are given in Eqs. (1) and (2), respectively [25, 26]. In these expressions, *V*_*π*_, *e*, and *a* are represented as carbon-carbon bond energy, electronic charge, and lattice constant, respectively. The structure of the CNTFET is illustrated in Fig. 1.

1 $$\:{V}_{th}=\frac{a{V}_{\pi\:}}{\sqrt{3}e{D}_{CNT}}$$

2 $$\:{D}_{CNT}=\frac{a\sqrt{{n}^{2}+{m}^{2}+nm}}{\pi\:}$$

### Power metrics

According to Eq. (3), the two primary sources of total power consumption (*P*_*T*_) in digital circuits are dynamic power (*P*_*D*_) and static or leakage power (*P*_*S*_). Static power comes from leakage currents even when the circuit is not in use, while dynamic power is used anytime a signal changes state at a circuit node. The largest contributor and most important factor in the total power dissipation of the digital systems is often dynamic power [10].

3 $$\:{P}_{T}={P}_{D}+{P}_{S}$$

The dynamic power dissipation inherent in digital circuits is principally comprised of two constituent elements: switching power and short-circuit power. Switching power is consumed when the circuit’s capacitances charge and discharge during signal transitions. Short-circuit power dissipation, conversely, arises during the brief interval when both the pull-up and pull-down networks are simultaneously active, thereby establishing a transient current path between the supply voltage (V_DD_) and ground (GND). The overall average power consumption can be expressed using Eq. (4) [10].

4 $$\:{P}_{T}={P}_{Switching}+{P}_{SC}+{P}_{S}$$

When the circuit’s capacitances charge and discharge during signal transitions, the switching power is consumed. A direct current path between V_DD_ and GND is established during short-circuit power dissipation, a phenomenon that occurs when the pull-up and pull-down networks are transiently active concurrently. Equation (4) is used to represent the overall average power consumption. The detailed discussion on switching power (*P*_*Switching*_*)*, short circuit power (*P*_*SC*_*)*, and *P*_*S*_ is presented in [10]. The switching activity factor, denoted by α, represents the average number of logic transitions occurring at a circuit node per clock cycle and is a dominant contributor to dynamic power dissipation. For a digital node, α is defined as [31]:

5 $$\:{\upalpha\:}=\frac{{N}_{transitions}}{{N}_{total\:inputs}}$$

where *N*_*transitions*_ is the total number of 0→1 or 1→0 transitions observed at that node over all possible input combinations, and *N*_*total inputs*_ is the total number of input vectors applied. For a full adder with three inputs (A, B, C), there are 2³ = 8 possible input combinations. The switching activity factor for the Sum and Carry outputs is therefore computed by counting the number of output transitions between successive input states and normalizing by the total number of transitions.

In the context of adder design and power metrics, the alpha parameter (α) represents the switching activity factor. This factor is crucial for calculating the dynamic power dissipation of the circuit. The switching activity factor is defined as the average number of signal transitions a circuit node undergoes per clock cycle [12].

For a full adder, the switching factor is calculated as shown in Eq. 6. Degrading α at the Sum ($\:{\alpha\:}_{Sum}$) and *Carry* ($\:{\alpha\:}_{Carry}$) output nodes is the primary strategy of reducing the switching power. The overall switching activity ($\:{\alpha\:}_{circuit}$) of the AFA circuit is the combination of these factors [32]:

6 $$\:{\alpha\:}_{Circuit}={\alpha\:}_{Sum}+{\alpha\:}_{Carry}$$

In an approximate full adder, α is not only an output metric but also a design parameter. By modifying the truth table and simplifying logic paths of adder, the unwanted output transitions can be ignored, which helps to reduce the switching activity factor. This instantly improves the dynamic power consumption as stated in Eq. 7 [31]:

7 $$\:{P}_{dynamic=}\alpha\:{\bullet\:C}_{L}\bullet\:{VDD}^{2}\bullet\:f$$

The outputs of the adder, Sum and Carry, exhibit a switching activity factor of 0.234 that results in an overall circuit switching activity of 0.468. This reduction in α is achieved by controlling the transitions in non-critical input combinations through logic approximations. This significantly reduces the low power dissipation. Thus, α plays an important role in guiding the architectural decisions of the proposed approximate adder, which is a key reason to obtain superior energy efficiency compared to conventional exact adders and existing approximate designs. At the transistor level of implementation, the adoption of design styles such as static CMOS and PTL contributes to the reduction of short-circuit power, reduction of transistor count and reduction of the internal node capacitances [10].

### Error metrics

Approximate computing can be carried out at multiple levels of concept that include the algorithmic, gate and transistor levels. The basic principle of this approach involves the introduction of marginal reduction in output accuracy to achieve improvements in power consumption, operational speed and area efficiency, respectively. Evaluating the total efficacy of this technique necessitates a careful balance between computational accuracy and key circuit performance metrics. The several important error metrics relevant to this trade-off are briefly discussed in [10].

Error Distance (ED) is defined as the absolute arithmetic difference between the exact and approximate output values for a particular input. The MAE constitutes the highest ED value noticed across the input set. In contrast, the TED is computed as the summation of ED values for all possible inputs. The MED corresponds to the arithmetic mean of all individual EDs. Since the magnitude of the MED is inherently dependent on the operational bit-width of the circuit, a normalized metric called NED is employed to facilitate comparison across various designs. The NED is calculated by normalizing the MED with respect to the maximum possible output value of the circuit. For an n-bit adder that includes an input carry at the least significant bit, which is maximum value is given as $\:{2}^{n+1}-1$. The formal mathematical expressions for ED, MAE, TED, MED and NED are provided from Eq. (8) to (12), respectively [10, 21, 22].

8 $$\:MAE=maximum\left\{ED\right\}$$

9 $$\:ED=\left|{E}_{xr}-{A}_{pr}\right|$$

10 $$\:MED=\raisebox{1ex}{$TED$}\!\left/\:\!\raisebox{-1ex}{$M$}\right.$$

11 $$\:NED=\raisebox{1ex}{$MED$}\!\left/\:\!\raisebox{-1ex}{$N$}\right.$$

12 $$\:TED=\sum\:_{i=1}^{M}{ED}_{i}$$

Here, *E*_*xr*_ stands for the exact result, *A*_*pr*_ for the approximate result, and *N* for the greatest output value that the circuit may create.

## Considered approximate full adder for comparative analysis

This section thoroughly examines and assesses a number of cutting-edge AFA cells that were developed utilizing various logic approaches, taking into account both their logical functioning and transistor-level architecture.

Another AFA is designed in [23] using CNTFETs. A principal advantage of this design is its generation of error-free carry output, thereby effectively mitigating the propagation of inaccuracies to subsequent, more significant bit positions. Consequently, the design achieves the lowest relative error distance among all the contemporary AFA designs. The simulations demonstrate their better image quality metrics in the motion detection application. The electric instrument is also employed to assess the design area. A comprehensive FOM is employed for comparison purposes, as power consumption, delay, area and ED are critical performance metrics. The proposed design outperforms existing approaches by delivering 23.8% to 83.6% greater performance than existing designs based on this metric.

The paper [10] introduces an AFA cell that integrates pass transistor logic and static CMOS techniques. The cell is implemented and simulated using CNTFET technology. In order to assess its functionality, numerous HSPICE simulations were implemented at various ambient temperatures, output loads and supply voltages, respectively. The results demonstrate substantial enhancements over the most effective existing design, including 18% reductions in delay and 10% in energy consumption. The proposed cell’s robustness was further investigated by analyzing the impact of CNT diameter variations using Monte Carlo simulations. These simulations confirmed that the cell is tolerant to process variations. The design was demonstrated to be effective in practical scenarios through MATLAB-based evaluations of figure quality metrics in an image blending task at the application level.

A motion detection application-specific, low-leakage AFA cell that is highly stable and designed with top gate CNTFETs is presented in [13]. In low-power multimedia systems, energy efficiency is more important than absolute accuracy, and inexact arithmetic circuits have become more prevalent. The proposed design is assessed using both circuit-level metrics, including average power, propagation delay, energy, and leakage power, as well as application-level metrics. The AFA cell accomplishes a substantial reduction in leakage power and utilizes 89.2% less energy than the most recent AFA designs, while maintaining other performance parameters within acceptable limits according to the results. By demonstrating satisfactory image quality metrics in motion detection tasks, MATLAB-based experiments further validate its effectiveness. Furthermore, the evaluations carried out at different temperatures and supply voltages show that the circuit is stable and strong.

A highly accurate AFA cell that uses both CMOS and PTL types is explained in [19]. At the transistor level, CNTFET technology is used to accomplish design work. To thoroughly evaluate its effectiveness, detailed simulations were performed at both the circuit and application levels. The HSPICE tool is used to do simulations at the transistor level. The findings show that the proposed circuit improves the figure of merit metric by at least 12% compared to the most current state-of-the-art designs. The AFA cells have been integrated into the RCA architecture for application-level validation. This developed RCA is utilized in image processing algorithms to detect motion and edges. The evaluations of performance were conducted in MATLAB. The results of the simulation show that the generated images have good quality metrics. Finally, to find a balance between hardware efficiency and application-level performance, full figures of merit are developed. The proposed circuit offers substantial improvements in these parameters relative to current methodologies.

## Proposed approximate full adder; design and operation

The truth table in Table 1 is employed to create the proposed AFA circuit. The Sum and Carry outputs are not the same as the usual complete adder logic in three and one cases, respectively. The circuit has a switching activity of 0.2344 for each output and 0.4688 for the entire circuit. We observed the following key error metrics: an MAE of 1, a TED of 3, a MED of 0.375, and a NED of 0.125.

**Fig. 2:**
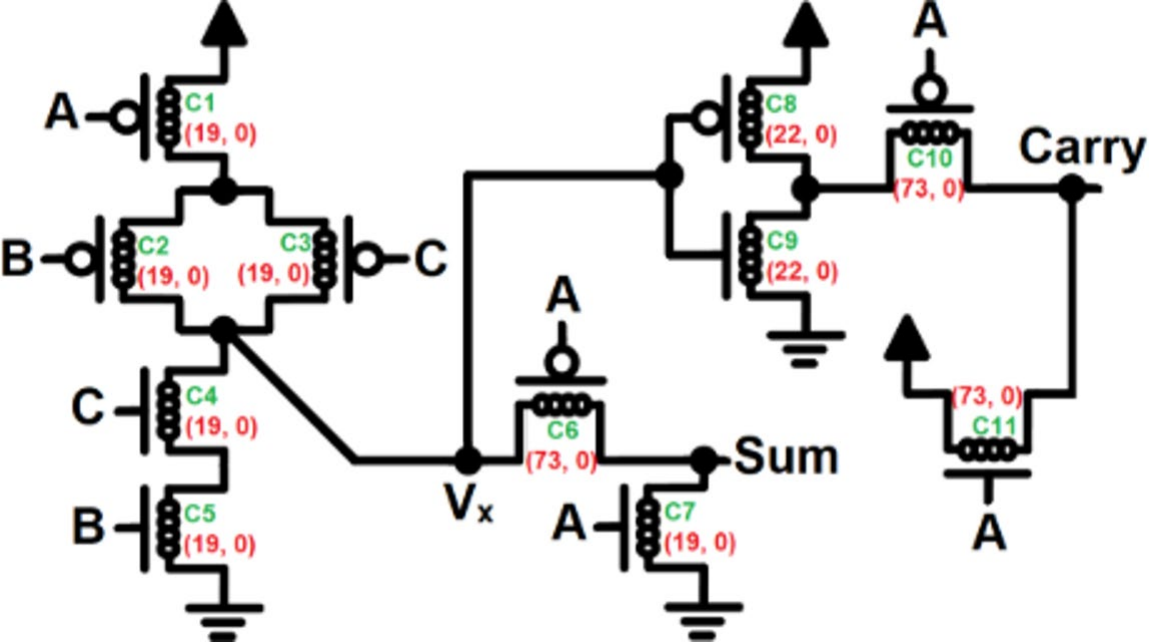
Proposed AFA circuit, designed by CNTFETs

Figure 2 shows the transistor-level schematic of the proposed AFA circuit, which is developed using CNTFETs. There are a total of eleven transistors in the design: six p-type and five n-type CNTFET devices. The transistors C1 to C5 constitute a NAND gate, and the output (logic “1”) depends on input A. This setup minimizes the power use by reducing unnecessary switching at the internal node V_X_. Pass-transistor logic controls the Sum output. When A is at logic “0”, transistor C6 turns on and connects the Sum output to node V_X_. On the other hand, logic “1” at A turns on transistor C7, which sends the Sum output to ground. Similarly, the Carry output generation is governed by input A. A logic ‘0’ turns on C10, connecting the Carry output to the complement of V_X_. A logic ‘1’ activates C11, pulling the Carry output to V_DD_. To mitigate the V_th_ drop, pass transistors C6, C10, and C11 are designed with a larger CNT diameter, specified by a chirality vector of (73, 0). This yields a reduced V_th_ = 0.0752 V. Furthermore, to enhance the performance of the Carry output path, the inverter formed by transistors C8 and C9 is implemented with a chirality vector of (22, 0) with V_th_ = 0.25 V.

**Table 1:** Characterization of the proposed approximate full adder: truth table and error metrics

A	B	C	Exact		Approximate		ED
			Sum	Carry	Sum	Carry	
0 0	0 0	0 1	0 1	0 0	1 1	0 0	1 0
0 0 1 1 1 1	1 1 0 0 1 1	0 1 0 1 0 1	1 0 1 0 0 1	0 1 0 1 1 1	1 0 0 0 0 0	0 1 1 1 1 1	0 0 1 0 0 1

## Simulation results and discussions

The performance of the proposed AFA circuit was assessed through simulations performed in Synopsys HSPICE, utilizing the SPICE-compatible Stanford 32-nm CNTFET technology model [33–35]. Metrics for delay, power dissipation, and the power-delay product (PDP) were extracted under a 0.5 V supply voltage at 27 °C. Input signals with a frequency of 1 GHz and 10ps transition times were applied, with the output driving a fan-out of four (FO4) load.

To ensure an equitable analysis, the proposed circuit’s performance was compared with that of other AFAs from the literature, namely stacked leakage control approximate full adder (SLCAFA) [20], NAND-NOR-based approximate full adder (NNAFA) [23], energy-area efficient approximate full adder (EAEAFA) [10], bridge-based approximate full adder (BBAFA) [13], and highly-efficient approximate full adder (HEAFA) [19]-using the same simulation setup.

The circuit delay is the total propagation delay across all input-to-output paths. The average power is measured while the circuit functions with random input vectors to show the switching activity. Then, the PDP is calculated by multiplying delay and PDP together. This is an important metric for evaluating the energy-efficient overall design [36–40].

**Fig. 3:**
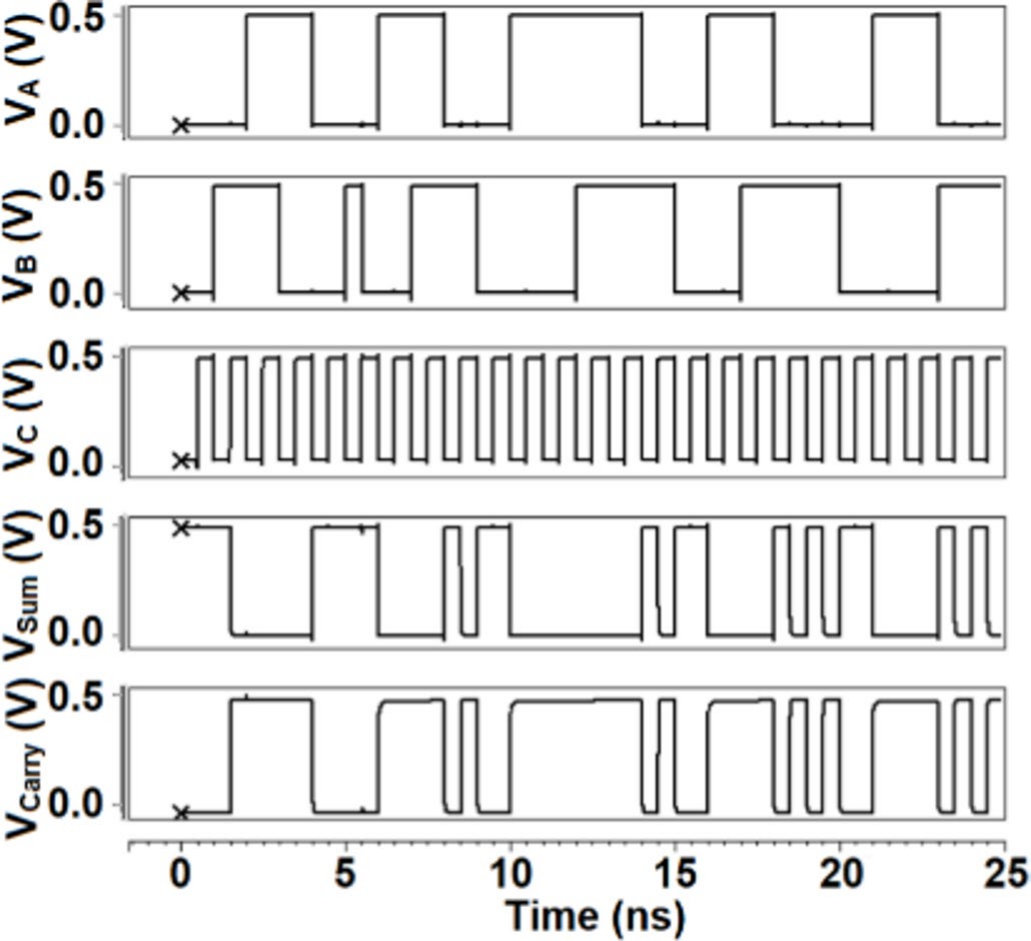
Transient response of the proposed AFA at normal operating conditions (V_DD_ = 0.5 V, Temp. = 27 °C)

The transient response in Fig. 3 is acquired under traditional operating conditions(VDD = 0.5 V, Temp. = 27 °C) confirms the circuit accurate functionality as presentedin truth table (see Table 1)

### Performance analysis of the proposed AFA

This section presents the performance analysis of the proposed AFA with a specific focus on three critical aspects, such as the functional importance of transistor C1, the impact of CNT chirality vectors selected for the inverter (transistors C8 and C9) and robustness against process, voltage and temperature (PVT) variations.

In nominal simulation conditions (V_DD_ = 0.5 V), the proposed AFA encounters a delay of 14.37ps, a power consumption of 30.13nW and a PDP of 0.433aJ, respectively. The transistor C1 was removed from the circuit, and a simulation was performed to know its role. The findings show a slight reduction in delay of 0.49%; however, this change increases the power to 1.25 times more. Therefore, the PDP is increased by 24.48%. The decrease in energy efficiency occurred due to transistor C1’s switching control being removed. This caused extra transitions at the internal node V_X_, which increased dynamic power dissipation. Table 2 shows a side-by-side comparison of the AFA’s performance with and without transistor C1.

**Table 2:** Functional importance of transistor C1 in the proposed AFA circuit

Metrics	With transistor C1	Without transistor C1	Change
Delay (ps) Power (nW)	14.37 30.13	14.30 37.71	* -*0.49% * +*25.16%
PDP (aJ)	0.433	0.539	* +*24.48%

**Fig. 4:**
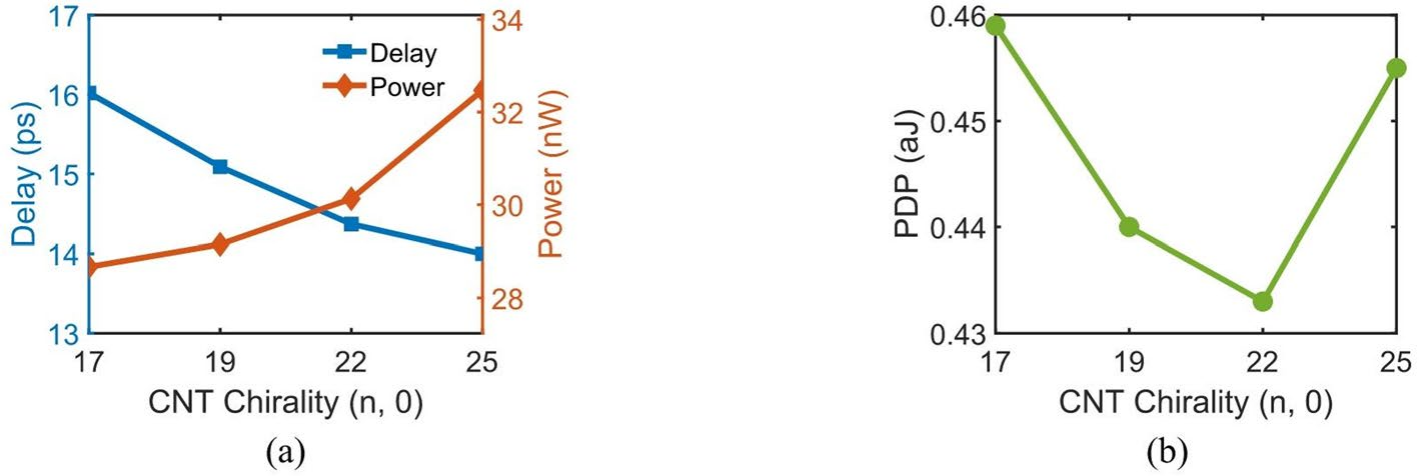
Effect of inverter’s CNT chirality (transistors C8 and C9) on the proposed AFA circuit performance. **a** Delay and power, and **b** PDP

The impact of the inverter’s CNT chirality for transistors C8 and C9 on the proposed AFA design performance is noticed in Fig. 4. The simulations are performed at V_DD_ = 0.5 V to explore the effect of varyling the chiral index *n* (with *m* fixed at 0) on delay, power and PDP, respectively. The results demonstrate that increasing *n* leads to a high delay but reduces the dynamic power consumption, a consequence of corresponding reduction in the bandgap and threshold voltage. This trade-off is accomplished in a non-linear relationship with PDP that initially decreases before increasing. The lower PDP representing the optimal balance between speed and energy consumption, is for CNT chirality (22, 0).

**Fig. 5:**
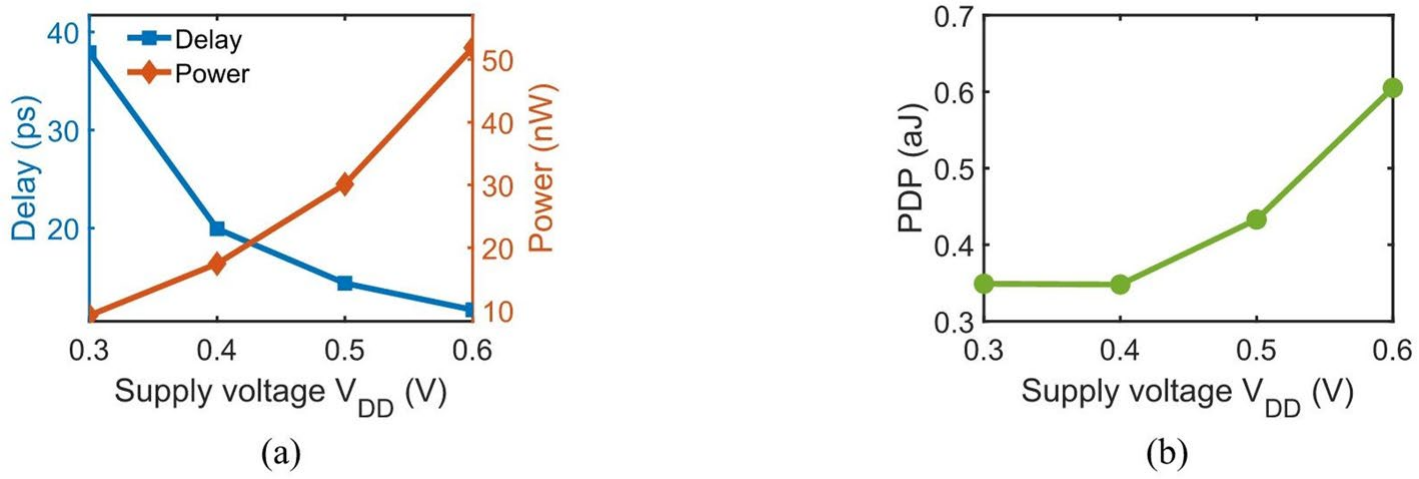
Effect of V_DD_ variation on the proposed AFA circuit performance. **a** Delay and power, and **b** PDP

Figure 5 shows the operational characteristics of the proposed TFA for various V_DD_ conditions. From Fig. 5(a), increasing V_DD_ value results in a significant reduction in propagation delay, which is attributed to the inverse relationship between switching speed and supply voltages. Concurrently, power consumption demonstrates a substantial increase due to the quadratic dependence of dynamic power on V_DD_. The combined impact of these opposing trends manifests as consistent elevation in the PDP, which is shown in Fig. 5(b).

**Fig. 6:**
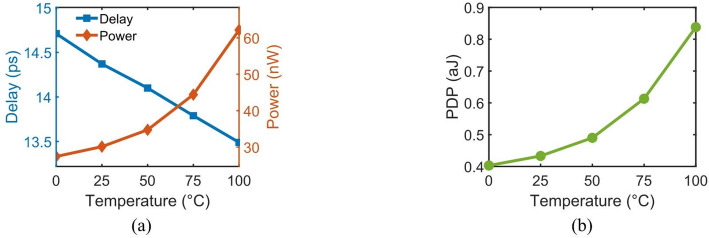
Effect of temperature variation on the proposed AFA circuit performance. **a** Delay and power, and **b** PDP

Figure 6 presents the performance of the proposed AFA under the temperature range from 0 °C to 100 °C. From Fig. 6(a), the elevated temperatures induce increased carrier scattering and degraded carrier mobility in semiconductor materials, resulting in degradation of delay. Concomitantly, power consumption increases significantly due to increased sub-threshold leakage currents, which exhibit exponential temperature dependence according to the Boltzmann relationship. The cumulative effect of these tendencies results in a gradual decline in PDP, as seen in Fig. 6(b).

**Fig. 7:**
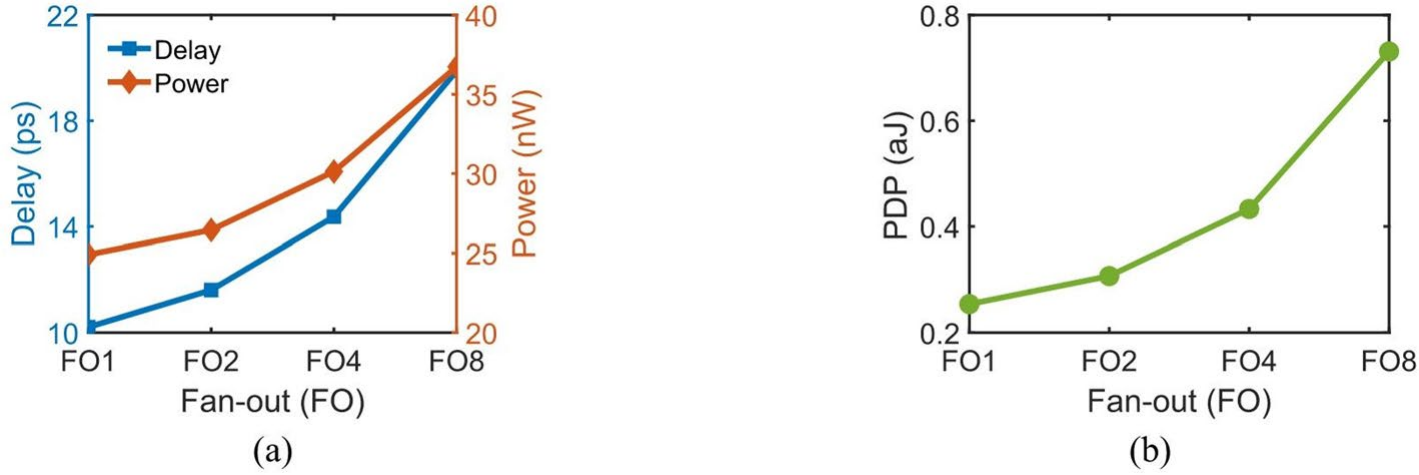
Effect of output load variation on the proposed AFA circuit performance. **a** Delay and power, and **b** PDP

Figure 7 shows the comprehensive performance investigation of proposed AFA under various capacitive loads. To consistently assess the load dependence, the outputs Sum and Carry are subjected to fan-out configurations of FO1, FO2, FO4 and FO8, representing increasing output capacitance values. The analysis shows that increased capacitive loads significantly decrease all three performance metrics. The propagation delay increases because of extended RC time constant required to charge and discharge higher output capacitances (see Fig. 7a). The power consumption increases with the increased switching energy dissipation per transition (see Fig. 7a). Therefore, the PDP exhibits considerable drop (see Fig. 7b), indicating that output loading conditions seriously impact both the speed and energy efficiency of proposed AFA design.

**Fig. 8:**
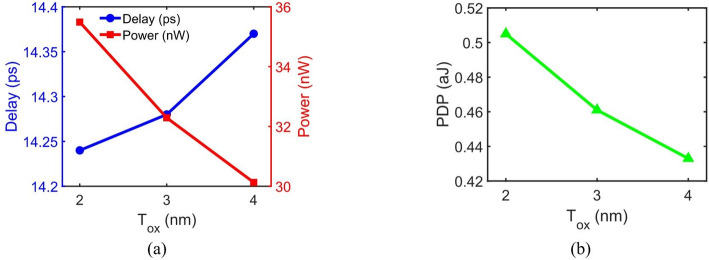
Effect of gate oxide thickness variation on the proposed AFA circuit performance. **a** Delay and power, and **b** PDP

Figure 8 presents the performance investigation of proposed AFA under various oxide thicknesses (Tₒₓ). It is noted that reducing the Tₒₓ from 4 nm to 2 nm results in a 1.1% enhancement in delay, attributed to improved gate control and increased gate capacitance. Conversely, the reduction in Tₒₓ increases the power consumption by 17.8% because of increased gate leakage currents and increased capacitances. Thus, the PDP shows a 16.6% reduction (see Fig. 8b), indicating that thinner oxides improve switching speed, but they compromise total energy efficiency because of disproportionate increases in power dissipation.

**Fig. 9:**
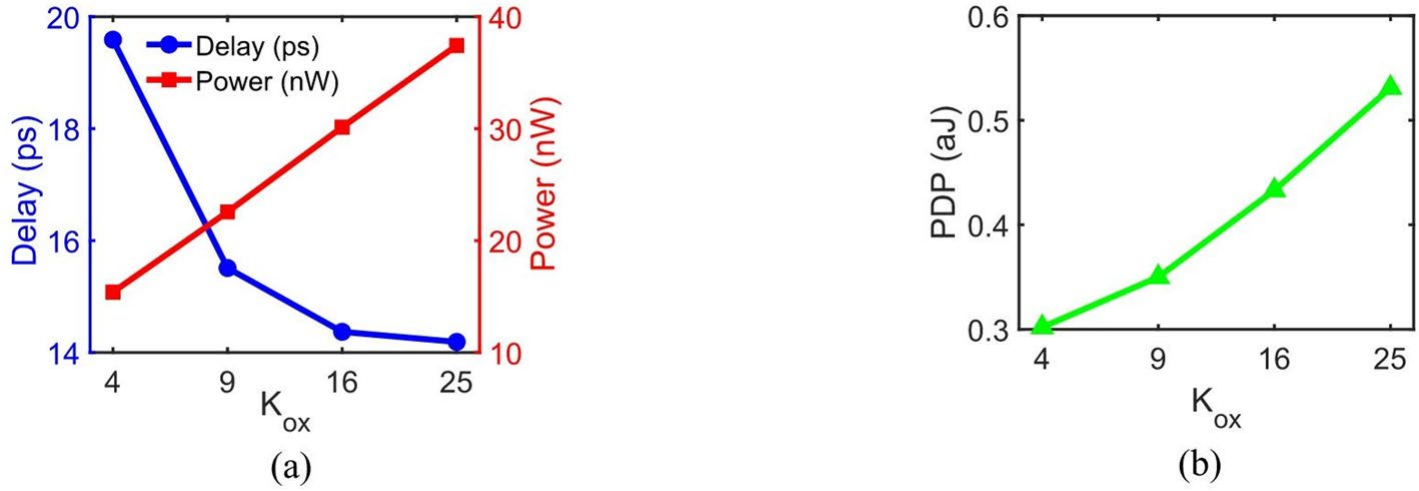
Effect of gate oxide dielectric constant variation on the proposed AFA circuit performance. **a** Delay and power, and **b** PDP

Figure 9 illustrates a performance analysis of gate oxide dielectric constant (K_ox_) variations on the performance metrics of AFA design. From Fig. 9(a), using high-K dielectric materials improves gate capacitance, resulting in enhanced gate control and reduced delay. Conversely, this increased gate capacitance elevates dynamic power because of greater switching energy requirements per transition. The contending impacts of the performance metrics yield an increase in PDP (see Fig. 9b), indicating that high-K dielectrics improve design speed, but they impose fundamental trade-offs in total energy efficiency.

**Fig. 10:**
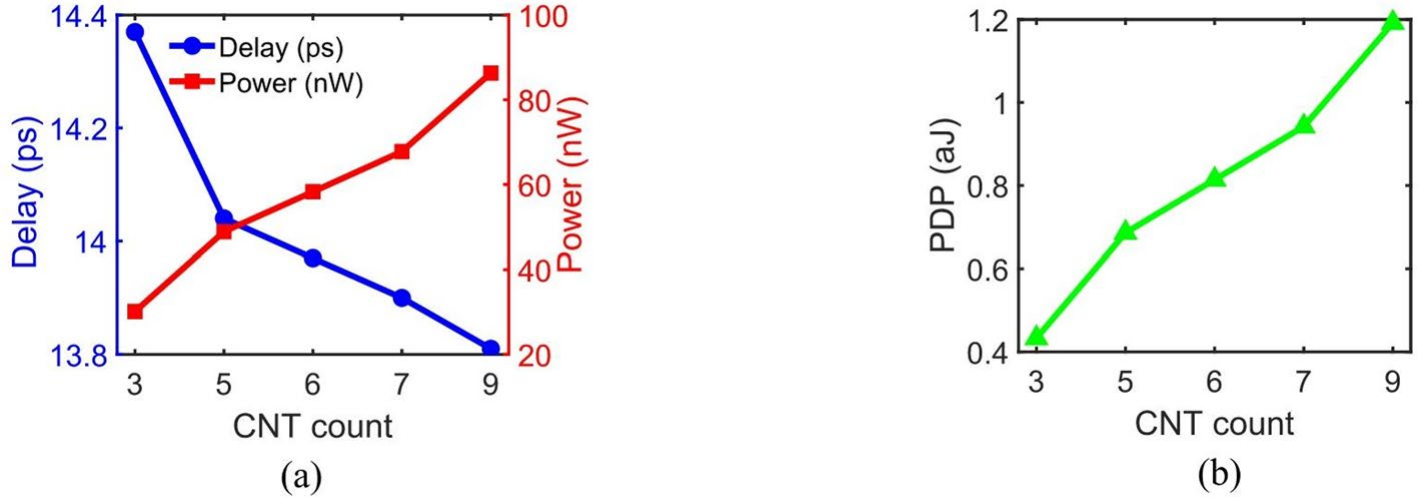
Effect of CNT count variation on the proposed AFA circuit performance. **a** Delay and power, and **b** PDP

Figure 10 analyzes the impact of CNT count variations on performances. Figure 10(a) illustrates that increasing the CNT counts from 3 to 9 reduces delay because of enhanced current drive capacity through the parallel conduction routes. On the other hand, the power consumption increases significantly owing to the increased gate capacitance and leakage currents. Figure 10(b) shows a corresponding decrease in PDP. The result emphasizes the important significance of optimizing CNT counts to achieve the balance between performance and power consumption in the proposed AFA design.

**Fig. 11:**
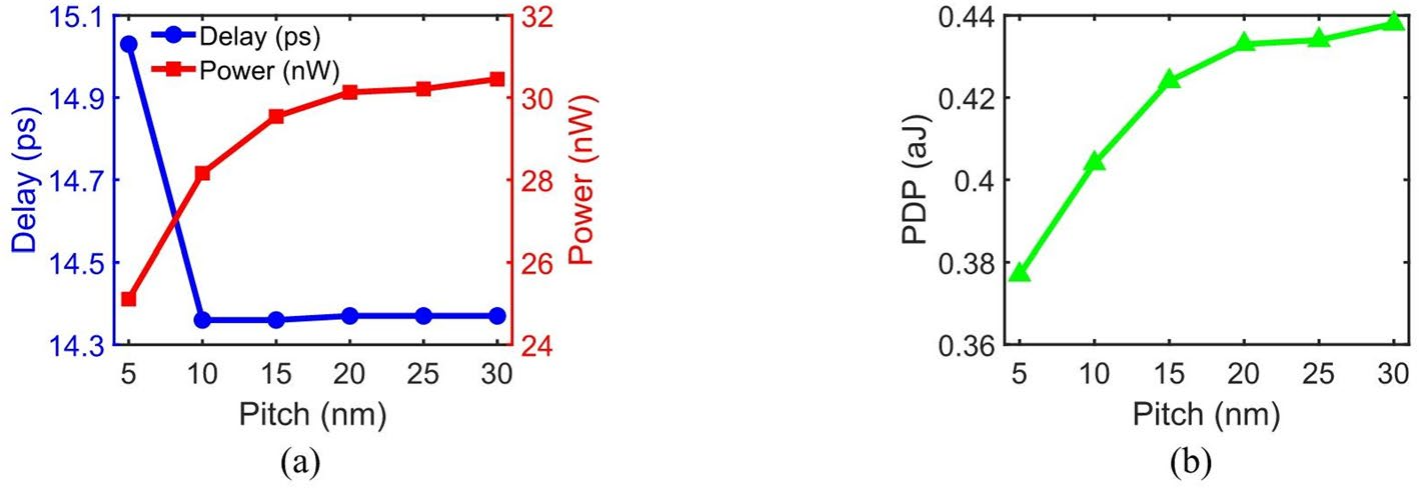
Effect of pitch variation on the proposed AFA circuit performance. **a** Delay and power, and **b** PDP

Figure 11 illustrates pitch variation impacts from 5 nm to 30 nm on the proposed AFA cell. As illustrated in Fig. 11(a), the delay improved by 4.5% when it scaled from 5 nm to 10 nm because of the reduced wire capacitance, but it remains constant beyond 10 nm range, indicating no further performance could be improved. Power consumption increases by 21.2% across the range because of improved electrostatic coupling effects. Therefore, Fig. 11(b) illustrates the PDP increases by 16.2%.

The analysis of CNTFET process variations such as channel dimensions, oxide thickness, CNT density, CNT diameter and pitch is important for optimizing AFA performances. This work utilizes Monte Carlo simulation with 1000 samples to illustrate the effect of these variations on delay, power and PDP, respectively [21, 33]. The simulation offers a robust performance with reduced standard deviation (*σ*)-to-mean (*µ*) ratios, confirming the circuit’s resilience to inherent process variations and ensuring reliable schematic operation.

**Fig. 12:**
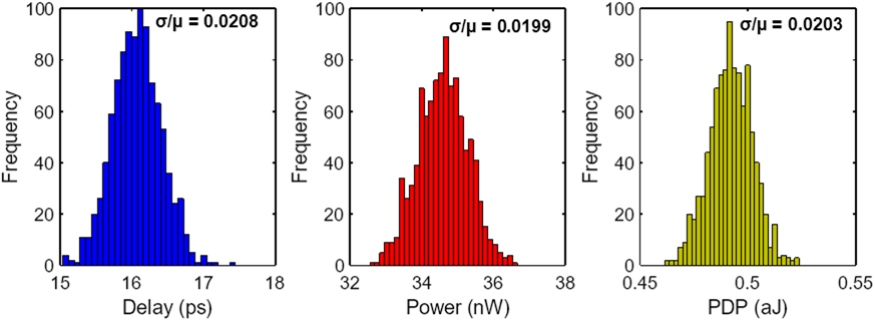
1000-run Monte Carlo simulation results of delay, power, and PDP of the proposed AFA cell

Depending on the findings from PVT variation investigations, we continued to optimize CNTFET device parameters. This procedure is essential to assure stable operation under various fluctuations. The resulting optimized parameters are placed in Table 3.

**Table 3:** Optimized parameters of the CNTFET device

Parameter	Brief description	Value
*L*_*ch*_	Physical channel length	32 nm
*L*_*dd*_, *L*_*ss*_	The mean free path in the intrinsic CNT	32 nm
*E*_*fi*_	The Fermi level of the doped S/D CNT for 0.8% doping level	0.6 eV
*K*_*gate*_	The dielectric constant of high-k top gate dielectric material (*HfO*_2_)	16
*T*_*ox*_	The thickness of the high-k top gate dielectric material	4 nm
*Pitch or S*	The distance between two adjacent CNTs within the same device	10 nm
*N*	The number of tubes in the device	3

### Performance comparison with prior works

The proposed AFA is evaluated under standard simulation conditions (V_DD_ = 0.5 V, Temperature = 27 °C) and compared to contemporary AFA architectures specifically SLCAFA [22], NNAFA [23], EAEAFA [10], BBAFA [13], and HEAFA [19] across various performance metrics like propagation delay, power consumption, PDP, switching activity factor and error characteristics, correspondingly. Table 4 shows the comparative performances of the AFA designs investigated.

The proposed design uses one extra transistor compared to EAEAFA [10] and three transistors compared to HEAFA [16], while featuring one fewer transistor than the remaining benchmark designs. The proposed AFA attains a 52.9% reduction in delay compared to SLCAFA architecture [22] and a 28.6% reduction compared to EAEAFA [10] while exhibiting 18.1%, 15.6% and 4.9% increases in delay over the NNAFA [23], BBAFA [13] and HEAFA [19] designs, correspondingly.

From the power consumption viewpoint, the proposed AFA design achieves the lowest power dissipation among all other architectures establishing 7.2% improvement compared to HEAFA [19] and 66.5% improvement compared to NNAFA [23], respectively.

Furthermore, the proposed AFA establishes superior energy efficiency, yielding the lowest PDP across all other designs. The architecture shows the PDP enhancements from 2.7 to 81.1% over the existing state-of-the-art AFA implementations.

The proposed AFA circuit exhibits switching activity factors for both Sum and Carry outputs that are comparable to those of SLCAFA [22] and EAEAFA [10] architectures, while demonstrating significantly reduced switching activity compared to NNAFA [23], BBAFA [13], and HEAFA [19] designs. Furthermore, the error characterization reveals identical MED and NED metrics between the proposed AFA design and both SLCAFA [22] and EAEAFA [10] implementations, indicating equivalent arithmetic accuracy performance among these architectures.

**Table 4:** Performance comparison between proposed AFA and prior works at V_DD_ = 0.5 V

Metrics	SLCAFA[22]	NNAFA[23]	EAEAFA[10]	BBAFA[13]	HEAFA[19]	Proposed
Transistor count	12	12	10	12	8	11
Delay (ps)	30.49	17.54	20.13	12.43	13.7	14.37
Power (nW)	75.16	89.89	44.42	72.96	32.45	30.13
PDP (aJ)	2.292	1.577	0.894	0.907	0.445	0.433
$\:{\alpha\:}_{Sum}$	0.234	0.25	0.234	0.25	0.25	0.234
$\:{\alpha\:}_{Carry}$	0.234	0.25	0.234	0.25	0.25	0.234
$\:{\alpha\:}_{Circuit}$	0.468	0.5	0.468	0.5	0.5	0.468
MAE	1	1	1	1	1	1
TED	3	2	3	2	2	3
MED	0.375	0.25	0.375	0.25	0.25	0.375
NED	0.125	0.083	0.125	0.083	0.083	0.125

**Fig. 13:**
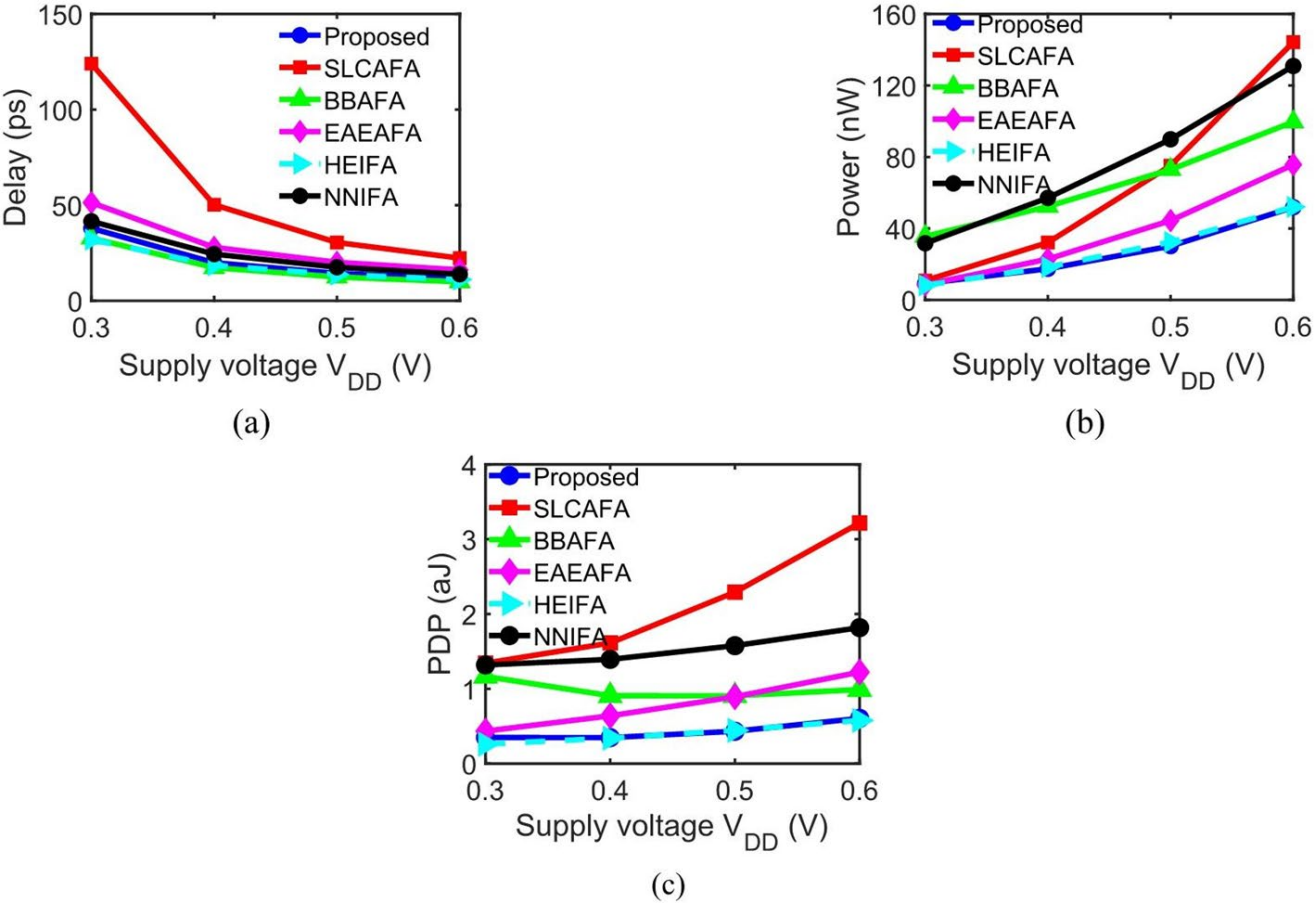
**a** Delay, **b** Power, and **c** PDP of the investigated AFA circuits against V_DD_ variations

The comprehensive performance evaluation of all investigated AFA circuits was conducted across multiple operating voltage levels, with the resulting delay, power consumption, and PDP characteristics presented in Fig. 13. The analysis demonstrates that the proposed AFA architecture exhibits superior power and energy efficiency characteristics not only at the nominal supply voltage of 0.5 V but also consistently maintains this performance advantage across the entire evaluated voltage range.

It is worth noting that the selected error metrics, namely Error Distance (ED), Maximum Error Distance (MAE), Total Error Distance (TED), Mean Error Distance (MED), and Normalized Error Distance (NED), are widely adopted standard metrics for evaluating approximate arithmetic circuits because they quantitatively measure the deviation between exact and approximate outputs. These metrics were selected to comprehensively analyze both the worst-case and average-case computational inaccuracies of the proposed approximate full adder. Specifically, MAE represents the maximum output deviation, TED measures the cumulative error across all input combinations, MED evaluates the average error behavior, and NED provides a normalized comparison independent of output bit-width. Therefore, these metrics enable a fair and comprehensive comparison between the proposed design and existing state-of-the-art approximate full adders. The significance of these metrics lies in their ability to evaluate the trade-off between computational accuracy and hardware efficiency, which is a critical requirement in low-power approximate computing applications. Furthermore, the correctness and practical applicability of the proposed approximate full adder have also been validated at the application level through image blending implementation using PSNR, SSIM, and figure-of-merit evaluations. The obtained image quality and efficiency metrics demonstrate strong agreement with the expected theoretical behavior of approximate arithmetic circuits for error-resilient applications.

## Image blending application

**Fig. 14:**
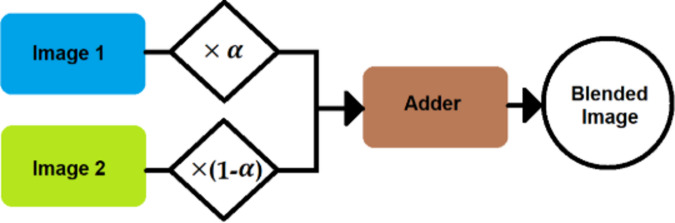
The procedure for image blending

In graphics and vision applications, image blending is very significant since it permits combining many images into one scene. Figure 14 shows the blending process which uses an alpha factor (*α*) to determine the degree of transparency level [19].

The process is stated in Eq. 13, where α lies in the range (0, 1) [20]. The MATLAB is used to conduct blending for $\:\alpha\:$ = 0.2.

**Fig. 15:**
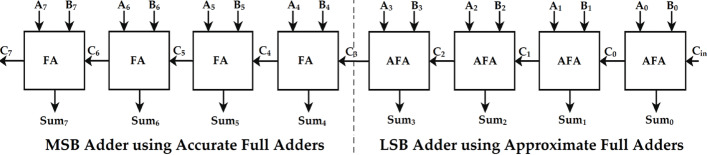
The block diagram of the 8-bit adder used for image blending

Figure 15 shows the 8-bit adder architecture that is used to build the system [38]. This design uses a hybrid adder structure that combines both approximation and exact computation elements. To take advantage of their better power and area efficiency, approximate full adders are assigned to the four least significant bits (LSBs). On the other hand, accurate full adders are used to make the four most significant bits (MSBs). The CNTFET-based complete adder shown in [41] is chosen as a reliable benchmark for performance study of the accurate modules.

**Fig. 16:**
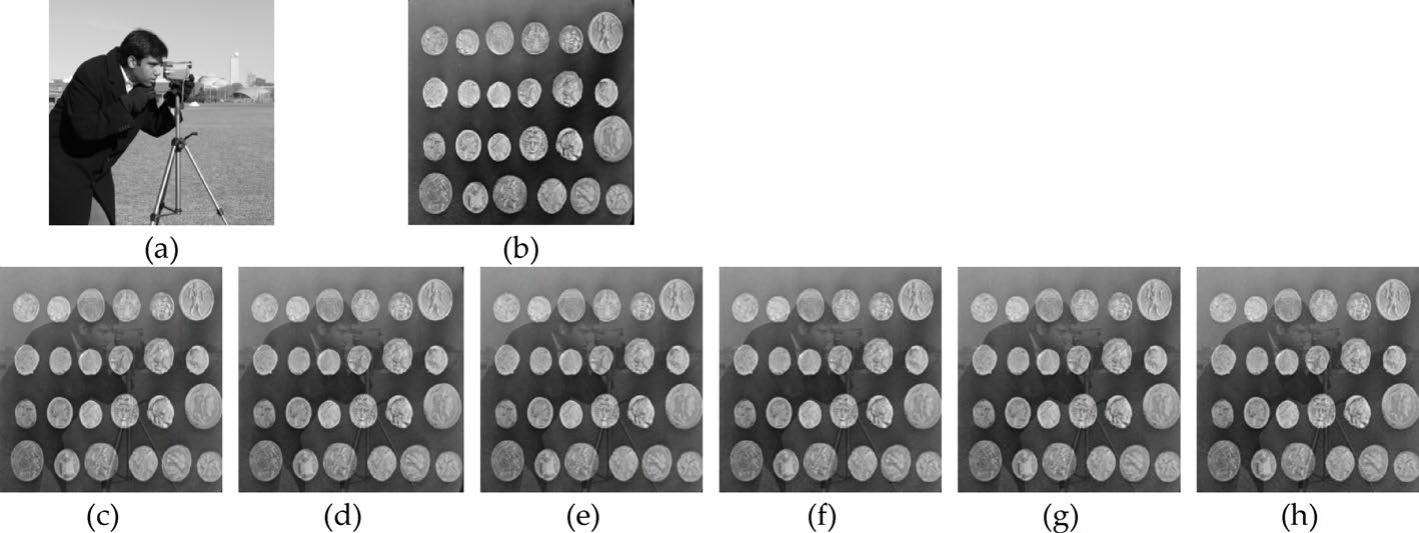
Image blending influence for $\:\alpha\:=0.2$. Input images: **a** Image 1 (photographer) and **b** Image 2 (coins). Blended images obtained from different AFA circuits: **c** SLCAFA [21], **d** NNAFA [23], **e** EAEAFA [10], **f** BBAFA [13], **g** HEAFA [19], and **h** Proposed

As illustrated in Fig. 16, the output images exhibit good visual quality for $\:\alpha\:$ = 0.2. The quantitative assessment relies on peak-signal-to-noise ratio (PSNR) [42] and structural similarity index (SSIM), respectively. A larger PSNR value reflects superior reconstruction quality of image. The SSIM ranges from 0 to 1, where 0 denotes no similarity between the images. The higher SSIM values correspond to high visual quality [43]. In addition, two FoMs, defined as the $\:\mathrm{F}\mathrm{o}\mathrm{M}1\:=\:\mathrm{P}\mathrm{D}\mathrm{P}/\mathrm{P}\mathrm{S}\mathrm{N}\mathrm{R}$ and $\:\mathrm{F}\mathrm{o}\mathrm{M}2\:=\mathrm{P}\mathrm{D}\mathrm{P}/\mathrm{S}\mathrm{S}\mathrm{I}\mathrm{M}$ that measure efficiency at the circuit level. The lower FoM values indicate superior designs [20]. The results of PSNR, SSIM and FoMs are listed in Table 5, confirming that the proposed architecture achieves competitive image quality with enhanced efficiency.

**Table 5:** The quality characteristics of blended images for $\:\alpha\:=0.2$

Metrics	SLCAFA [22]	NNAFA [23]	EAEAFA [10]	BBAFA [13]	HEAFA [19]	Proposed
PSNR (dB)	31.29	32.37	32.20	32.37	31.25	32.20
SSIM	0.7452	0.7885	0.7848	0.7885	0.7592	0.7848
FoM1	0.0733	0.0487	0.0278	0.0280	0.0142	0.0134
FoM2	3.0757	2	1.1391	1.1503	0.5861	0.5517

## Conclusions

This paper presents an approximation full adder cell designed for low power dissipation and outstanding energy efficiency, intentionally introducing logical imperfections in its *Sum* and *Carry* generation. The design is thoroughly analyzed at both the circuit and application level to determine its efficacy. A circuit-level simulation is performed in Synopsys HSPICE with 32-nm Stanford CNTFET SPICE model. The simulation results are carried out with a supply voltage of 0. 5 V and temperature of 27 °C, respectively. The results demonstrate that the proposed cell exhibits a propagation delay of 14.37ps, average power consumption of 30.13nW, and PDP of 0.433aJ, correspondingly. The comparative analysis shows that the proposed design achieves a power reduction between 7.2% and 66.5% and an energy efficiency improvement between 2.7% and 81.1% over the existing designs at the expense of a marginal delay penalty. At the application level, the cell is distributed in an image processing operation (i.e., image blending). The output quality is determined using the PSNR and SSIM with results indicating acceptable visual fidelity. The overall efficiency of the proposed design is further validated through the definition of two application-specific FoM. Future research will explore its integration into neuromorphic architectures, specifically evaluating its impact on the learning precision and energy efficiency of spiking neural networks, alongside a rigorous characterization of its resilience to process, voltage, and temperature (PVT) variations.

## Data Availability

All data generated or analyzed during this study are included within this article.
